# Ground Breaking and Breaking Ground

**DOI:** 10.1100/tsw.2001.5

**Published:** 2001-02-02

## Abstract

The Howard Hughes Medical Institute’s decision to build a home for wayward interdisciplinary scientists topped the news from Nature this week. Science led with shaky predictions and dire warnings from the Indian subcontinent.

*Nature* leads off the week with a story about the Howard Hughes Medical Institute's (HHMI) plans to build a plush $500 million suburban campus in Chevy Chase, Md. (*Science* also covered this story in its second slot.) According to both magazines, HHMI is primarily a funding source for a network of researchers scattered throughout the world. But when the HHMI decided to cap the number of researchers at 350, they found they had money to spare. Saving it is out of the question, says *Nature*, because HHMI is required by law to spend 3.5% of their endowment every year. So HHMI will spend the cash stocking the new center, which is scheduled to open in 2005, with high-tech goodies such as bioinformatics software and low-temperature electron microscopes. Scientists will then be invited for multi-year stays to play with the toys at the "scientific hotel," report both journals. The institute will also serve as an incubator for groups doing cutting-edge research in hot fields such as proteomics and bioinformatics. It all sounds very profitable, but HHMI vice president for biomedical research Gerry Rubin says, in *Science*, "We are not doing this as a way to increase our endowment."

Although they could never have predicted exactly when and where it would happen, the devastating earthquake that killed approximately 10,000 people last week in India came as no surprise to geologists, reports *Science* in their lead story this week. And worse quakes are almost certainly on the way. The article quotes tectonophysicist Peter Molnar of the University of Colorado, Boulder, who ominously said, "This is a wake-up call, but it isn't as big as the quake that will come." Other scientists interviewed by *Science* agreed and offered a grim forecast: random massive quakes will erupt across India throughout the coming decades. As usual, the culprit is plate tectonics. For at least the last 150 million years, says *Science*, the Indian subcontinent has been slowly plowing into Asia, driving the Himalayas into the sky. Conventional wisdom holds that quakes should occur along the Indian-Asian fault line, and they do, but the same tectonic pressure has also weakened the crust under the entire subcontinent, creating weak spots that are vulnerable to earthquakes. This isn't just the idle speculation of tectonic theorists, either. Last week's Bhuj quake, reports *Science*, is the latest, and most deadly, in a string of six intraplate quakes that have shaken India in the last 50 years. *Nature* did not cover this story.

And in the latest genome news, Syngenta, a Swiss agrochemical company, has finished mapping the rice genome, report both *Science* and *Nature*. But if you want a copy, you will have to pay. A publicly available copy is in the works, but the Japanese group leading the international effort to create a public rice genome doesn't expect to finish for another three years. The wait may be worth it. Although Syngenta claims their map is "99.5%" complete, reports *Nature*, the scientists interviewed by *Science* seemed more skeptical. "Their coverage is very good, and it's certainly a lot of sequence, but it's still very far from a complete sequence," said Rob Martienssen of Cold Spring Harbor Laboratory in New York, a member of the international consortium.

This Week in Nature and Science, February 2, 2001
© 2001 TheScientificWorld, Inc.
